# Life threatening illness in popular movies-a first descriptive analysis

**DOI:** 10.1186/2193-1801-3-411

**Published:** 2014-08-05

**Authors:** Laura Drukarczyk, Carsten Klein, Christoph Ostgathe, Stephanie Stiel

**Affiliations:** Department of Palliative Medicine, University Hospital Erlangen, Friedrich-Alexander-Universität Erlangen-Nürnberg, Krankenhausstrass 12, 91054 Erlangen, Germany; Comprehensive Cancer Center CCC Erlangen-EMN, University Hospital Erlangen, Friedrich-Alexander-Universität Erlangen-Nürnberg, Erlangen, Germany

**Keywords:** Film, Terminal illness, Palliative care, Systematic search

## Abstract

In the last two decades, public attention towards illness, dying and death has evolved. In particular, advance care planning, living wills, end-of-life care, and autonomy are increasingly discussed. How this change in public awareness has influenced the presentation of dying and death in cinema needs clarification. Over a one year period, November 2011 until October 2012, a systematic search was conducted to identify movies dealing with incurable diseases produced in 1991–2010 35 movies could be identified and were analyzed in detail and investigated the presentation of illness and death. The number of movies focusing on terminal illness, dying, and death has increased since 1991. The total number of movies that made the yearly German Federal Film Board (FFA) hit list and included a focus on terminal illness, dying, and death increased from 1991 (1 movie) to 2011 (6 movies). The gender of the main characters suffering from terminal illness was distributed equally; three movies portrayed terminally ill children. More than one third of the terminally ill characters died in hospital. The terms “palliative” or “hospice care” were not mentioned once in any films. The number of movies dealing with terminal illness continues to increase and a considerable audience has shown interest in these films. Due to a limited true-to-life performance in the films, a presentation closer to reality could be a major public educational resource.

## Background

In the last two decades, parallel to the dynamic development of hospice and palliative care in Europe and other western societies, public attention towards illness as well as towards dying and death may have been increased. In particular issues of advance care planning, living wills, end-of-life care and autonomy have been discussed. It is unclear if and in how far this development in public awareness might have influenced the presentation of dying and death in cinema.

A recent article from an influential German Newspaper in regard to the American movie “Restless” and its depiction of a young girl dying from cancer stated that there may be a trend in the movie industry to focus more on issues of cancer and dying and death. The article was headed by the phrase “Dying for All the World to See” (Vahabzadeh 2011). Recently, the movie “Amour (Liebe)” produced by Michael Haneke depicting the trajectory of an older woman experiencing a stroke won the Academy Award for “Best Foreign Language Film of the Year” in 2013 (BR and WDR 2013). Finally, her husband chooses to be alone in taking care of his wife and at last to suffocate her with a pillow after the suffering due to the illness had seemingly become unbearable (to both).

The authors’ recognition of the issues incurable illnesses and dying and death in modern media has led to the motivation to investigate the handling of these topics in popular movies.

So far, little research on issues of handling terminal illnesses and death in movies has been performed so far, and the research that has been done is qualitative in nature. In a chapter entitled, “Cinematic Visions of Dying” from her book, “The Study of Dying”, McInerney qualitatively describes individual presentations of death in film (Kellehear 2009). This chapter outlines the dying process as it has been depicted in the cinematic roles of dying mothers, fathers, and homosexuals.

The chapter, “End of Life and Right to Die” published in “The Picture of Health,” issues concerning death, such as living wills, procedures in case of brain death, and physician assisted suicide, are explained on the basis of film scenes. specific details, such as advance directives and their juridical consequences are defined for laity (Colt et al. 2011) using film scenes for illustration.

Part four of “Bioethics in the Movies” (Shapshay 2009) offers a relational perspective, wherein the relationships and communications between performers are analyzed, showing how death is handled in selected movies.

In contrast to previous studies of terminal illness in film, which have been mostly qualitative in nature, the study presented here concentrates on frequency with regard to the variety and course of diseases, medications, symptoms, therapies, survivals and deaths.

In summary, former examinations of selected movies do not offer valuable clues to presentation of incurable diseases which is why the authors decided to carry out this investigation for gaining a first empirical overview of depicting cureless illnesses and its trend.

Movies are part of the cultural identity of a society and may depict, encourage, or foil societal developments. Movies serve as a didactic element for the transfer of knowledge and attitudes to the public at large. Likewise, movies that depict terminal illness may influence students of medicine (Klemenc-Ketis and Kersnik 2011) and the practice of educating medical staff on the spiritual components of death (Jung 2012). National and international movies play an instrumental role in explaining end of life issues and supporting their audience’s exploration of these issues. Recent developments in the use of internet-based platforms like Facebook provide a place to share knowledge and experiences, and thereby facilitate support for patients and their relatives (Smith 2011). As time progresses, further increases the relevance of these social platforms as tools for dealing with terminal illnesses. Yet, even though social media is gaining in popularity, currently, film remains a very popular medium, and this is especially true for many senior Germans. A German statistic on cinema visitors showed that the proportion of older generations going out to the movies increased whilst the number of younger people decreased steadily (FFA 2011). Consequently, the production and presentation of movies dealing with end-of-life issues and terminal illnesses could well match the interests of these older visitors.

### Study aim

To get a first empirical insight, a systematic movie search and quantitative analysis was performed. This study aimed to evaluate whether international movies dealing with terminal illnesses increased in German viewership during the last two decades and attempts to analyze how the issues of illness and death are presented in movies.

## Methods

A systematic movie search was started in January 2012 using the database of the German Federal Film Board (FFA) (Filmförderungsanstalt 2013). The FFA ranks the 100 most seen/visited cinema movies per year in Germany in so-called hit lists. Yearly, one list on international films and one on German productions are published. These lists of international productions (1986–2011) and German productions (1994–2011) were screened by analyzing every available summary on http://www.cinema.de, http://www.moviepilot.de and http://www.filmportal.de up until February 2012.

Inclusion criteria: national and international movies, that were shown in the cinema; movies popular in Germany (listed in the 100 most seen/visited cinema movies per year in Germany); movies, that deal with dying; movies with a main character suffering from a potentially terminal illness. Exclusion criteria: Movies not listed in the in the 100 most seen/visited cinema movies per year in Germany movies unavailable in German; movies dealing with the terminal illnesses of supporting actors.

All movies that met the inclusion criteria were ordered on DVD and analyzed quantitatively. Therefore, a standardized and structured data extraction tool was developed to include data on gender, age, smoking and drinking habits, illness, therapy, medication, reference to hospice and palliative care, symptoms, survival, care-giver reaction, place of death and cause of death. During data analysis, more attributes than were originally included in the standardized data extraction tool appeared to be relevant and were added. Each movie was then viewed a second time to review all new items.

Data-extraction was performed by one researcher (LD). In case of uncertainty about how to allocate an item, a second researcher (SS) was consulted and the item was discussed until agreement was achieved. Additionally, data from the FFA on the year of production, producing country, genre, director, distribution companies, number of viewers, and the rating on the hit lists were assigned to the data base. By October 2012 the data extraction was completed and the statistical analyses were carried out with SPSS 19.0 for Windows. To investigate probable changes over the time, some results were divided and compared for two time frames.

## Results

### Inclusion/exclusion of movies

**Figure 1:**
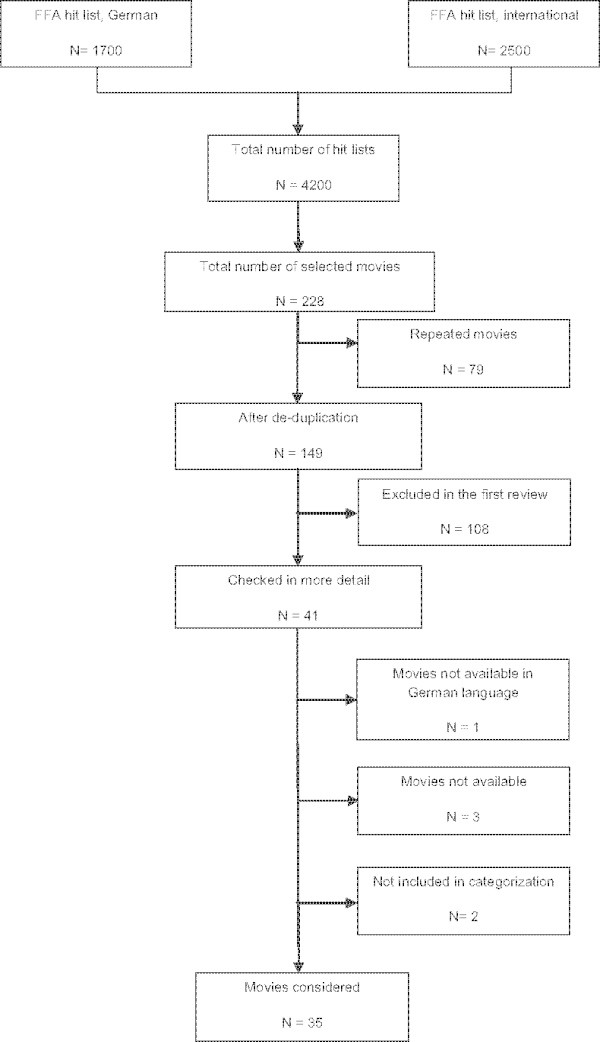
**Inclusion and exclusion of systematic film search; (FFA = German Federal Film Board).**

A total of 4200 movies were identified for first assessment. After screening the summaries, 3972 movies had to be eliminated for lack of subject matter relevant to the key issue of dying and death. After de-duplication of another 79 movies, 149 movies were evaluated in more detail. In a second review, 108 were excluded because the terminal illness portrayed in the film did not concern the main character. Finally, from the remaining 41 movies, four were not available. Two more had to be excluded because the story was incongruous to this study’s aim. One movie was excluded because the disease was suspected as a probable diagnosis, but not confirmed within the plot. The second was excluded because, although a terminal illness was mentioned, it was not developed in the movie. A total of 35 movies were analyzed with the data extraction tool (Figure 1, Table 1).

**Table 1:** **List of analysed movies (N = 35)**

Original title (German title if available)	English translation
Love and other drugs	Love and other drugs
Seven Pounds (7 Leben)	Seven Pounds
My Sister’s Keeper (Beim Leben meiner Schwester)	My Sister’s Keeper
The Bucket List (Das Beste kommt zum Schluss)	The Bucket List
Le scaphandre et le papillon (Schmetterling und Taucherglocke)	Butterfly and Diving Bell
Emmas Glück	Emma’s Bliss
Something’s Gotta Give (Was das Herz begehrt)	Something’s Gotta Give
Sweet November	Sweet November
Autumn in New York (Es begann im September)	Autumn in New York
Magnolia	Magnolia
Stepmom (Seite an Seite)	Stepmom
The Rainmaker (Der Regenmacher)	The Rainmaker
Knockin’ on heaven’s door	Knockin’ on heaven’s door
Kirschblüten Hanami	Cherry Blossoms Hanami
Marvin’s Room (Marvin’s Töchter)	Marvin’s Room
One Night Stand	One Night Stand
Boys on the side (Kaffee, Milch und Zucker)	Boys on the side
Muriel’s Wedding (Muriel’s Hochzeit)	Muriel’s Wedding
Philadelphia	Philadelphia
Les nuits fauves (Wilde Nächte)	Wild Nights
Dying young (Entscheidung aus Liebe)	Dying young
Le Havre	Le Havre
Satte Farben vor schwarz	Saturated Colors before Black
Das Ende ist mein Anfang	The End is my Beginning
Halt auf freier Strecke	Stop between Stations
Geliebtes Leben	Beloved Life
Same same but different	Same same but different
Drei	Three
Eine andere Liga	Another League
Almost heaven	Almost heaven
Fickende Fische	Fucking Fish
Grüne Wüste	Green Desert
Das Leben ist eine Baustelle	Life is a Building Site
Contagion	Contagion
Je n’ai rien oublié	Small World

### General information on movies

The total number of movies that made the yearly FFA hit list and included a focus on terminal illness, dying, and death increased from 1991 (1 movie) to 2011 (6 movies), whereas the average number of viewers per these movies has decreased (first time frame 932.055, second decade 468.080). A wide range of distribution companies such as Constantin Film™, Concorde™, Sony™ etc. published these movies, but no distributor appears more often than in five productions. The number of German productions increased from three in first time frame to 12 in the following decade. The inverse of this trend applies to US films. The number of American movies decreased during the period under review from 10 films to 6; four of the analyzed movies were produced in countries other than Germany or America (Table 2).

**Table 2:** **General information on the movies (N = 35)**

Characteristics of movies (N = 35)		N	%
**Producing Country**	USA	16	46
	Germany	15	43
	France	3	9
	Austria	1	3
**Genre**	Motion Picture	15	43
	Drama	14	40
	Comedy	6	17
**Main Theme**	Love	17	49
	Family	9	26
	Friendship	4	11
	Career	3	9
	Disaster	1	3
	Sports	1	3

### Patient characteristics

Gender of main characters was distributed equally. Three movies focused on terminally ill children. Patients were predominantly non-smokers (57%), had solid relationships and lived in stable social environments (66%). Female main characters had a survival rate of 75%, whereas survival rate in male was 31% (Table 3). Cancer (49%), AIDS/HIV (20%) and diseases of the cardiovascular system (6%) were the most commonly portrayed terminal illnesses (Table 4). Regarding the distribution of different diseases between 1991–2001 and 2002–2011, the first time frame evaluated in this study, cancer (67%) and AIDS/HIV (33%) are the only terminal illnesses considered in the movies. In the second decade, cancer is still the most frequent theme (45%), followed by AIDS/HIV (15%); however, other diseases (Table 4) became more prevalent in movies.

**Table 3:** **Patient characteristics (N = 35); differences to 100% due to round-off errors**

Characteristics of terminally ill main characters (N = 35)		N	%
**Age group**	Adult	32	91
	Children	3	9
**Smoker**	Yes	15	43
	No	20	57
**Consuming alcohol**	Yes	28	80
	No	7	20
**Disease’s Influence on Patients’ Career**	Yes	4	11
	No	12	34
	Unknown	19	54
**Survival (N = 18)**	Men (N = 16)	5	31
	Woman (N = 16)	12	75
	Children (N = 3)	1	33

**Table 4:** **Dying and death, symptoms and distress (N = 35); differences to 100% due to round-off errors**

Characteristics of dying and death of main characters in movies (N = 35)		N	%
**Primary Disease**	**Adults**		
	Cancer	17	49
	AIDS/HIV	7	20
	Unknown	2	6
	Cardiovascular Diseases	2	6
	Locked-in-syndrome	1	3
	Dementia	1	3
	Parkinson	1	3
	Fake Disease (MEV1)	1	3
	**Children**		
	Leukemia	2	6
	AIDS/HIV	1	3
**Entity of Cancer (N = 17)**	Undefined	5	29
	Leukemia	3	18
	Pancreatic Cancer	2	12
	Bone Cancer	1	6
	Prostate Cancer	1	6
	Breast Cancer	1	6
	Non-Hodgkin-Lymphoma	1	6
	Brain Tumour	1	6
	Neuroblastoma	1	6
	Brain and Lung Cancer	1	6
**Mentioning of the Disease**	Yes	30	86
	No	5	14
**Showing symptoms**	Yes	31	89
	No	4	11
**Therapy Measures**	Yes	26	74
	No	9	26
**Therapy (Multiple Answers Possible)**	Chemotherapy	8	20
	Surgery	4	11
	Consultation	3	9
	Intravenous Drip	2	6
	Irradiation	1	3
	Reanimation	1	3
	Puncture of Bone Marrow	1	3
	Organ Donation	1	3
**Medication**	Yes	30	86
	No	5	14
**Suffering**	Yes	29	83
	No	6	17
**Mentioning palliative or hospice care**	Yes	0	0
	No	35	100
**Survival**	Yes	18	51
	No	17	49
**Hospital Stay**	Yes	23	66
	No	12	34
**Place of Death (N = 17)**	Hospital	6	35
	Home	5	29
	Unknown	5	29
	Lakefront	1	6
**End of Life (N = 17)**	Death of Natural Causes of Disease	13	76
	Physician-Assisted Suicide	3	18
	Suicide (Attempt)	1	6
**Family Reaction**	Support	23	66
	Unknown	6	17
	Lack of Understanding	3	9
	Ignorance	2	6
	Denial	1	3
**Hospital Staff Reaction**	Care	31	89
	Disinterest	2	6
	Insensitive Behaviour	2	6
**Homecare**	Yes	27	77
	No	8	23

### Dying and death, symptoms and distress

**Figure 2:**
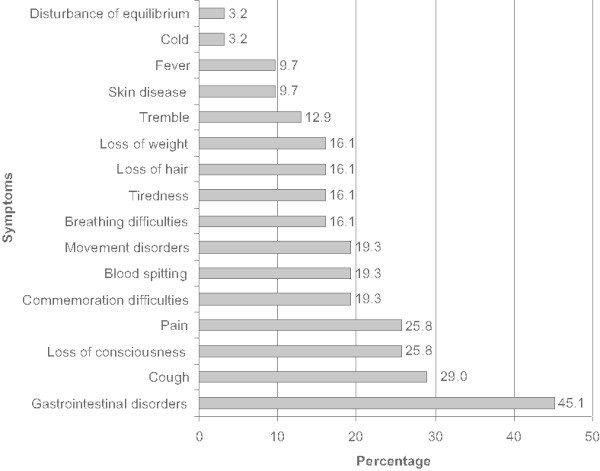
**Percentage (%) of symptoms occurring in movies (N = 35); (multiple answers possible).**

At least one symptom of a terminal illness was shown in 89% of movies. Most commonly gastrointestinal disorders (45%) and cough (29%) were observed. Additionally, pain and loss of consciousness were shown in a fourth of the movies and movement disorders, blood spitting and commemoration difficulties were seen in a fifth of all main characters (Figure 2). In 11% of the films no symptoms were presented. In 83% the main characters suffered recognizably from their diseases, whereas 17% showed no signs of distress. More than one third of the main characters died in a hospital, nearly another third died at home, one at a lakefront, and the place of death of almost another third remains unknown. The terms “palliative or hospice care” were not mentioned once in any context. The majority of the protagonists were supported by their family and 77% of the characters had somebody who took care of them (Table 4).

## Discussion

To our knowledge, this is the first descriptive analysis on the frequency of terminal illnesses with regard to variations and course of diseases, medication, symptoms, therapy, survival and death in popular national and international movies. Compared to the total amount of movies reviewed, only a rather small number of films reflect on dying and death. Nevertheless, over time this number is slowly increasing. The composition of current movie audiences includes more older people (FFA 2011). Younger people in the USA tend to go to the cinema less often. Experts argue that this may be in part due to the ongoing financial crisis (Rieger 2011). The same trend has been observed in Germany (FFA 2011). Another cause may be the broad availability of home entertainment systems and access to the newest cinema movies via a wide range of online movie platforms at any time straight from home. This trend might be a reason for producers to concentrate on the older viewers and adapt their movie topics to a more serious audiences’ mind.

In addition to the analysis of general information on the movies, several aspects of the main characters were analyzed. Interestingly, smoking and drinking characters were portrayed considerably more often than what would be representative of the general population. An even more interesting and disconcerting insight is that these smoking and drinking characters tended to have the better survival prognosis. This fact which contradicts real world health care facts may have a negative educational impact on the public.

Regarding characteristics of dying and death of main characters in movies, a rather broad spectrum of diseases was portrayed in film; but, their prevalence does not mirror clinical reality. This may be an issue of time and public awareness. In the 1990s AIDS/HIV was a major health care and public concern. So, not surprisingly, the films reviewed from that period featured only cancer and AIDS/HIV. Today, HIV and AIDS are less predominant in the stories. One possible explanation could be the progress in therapy for HIV patients and the overall improvement in survival rate. This diagnosis is not an “inescapable death sentence” anymore (Arbeitskreiswelt-Welt-AIDS-Tag 2009). This improved prognosis made the subject less dramatic and likely contributed to a significant loss in public interest, at least in the western societies. The trend towards cancer as a key issue in recent movies might be due to the fact that cancer incidence is still increasing (Statistisches Bundesamt Todesfälle 2012). Although cancer remains the most common disease in popular movies, a significant increase in the variation of diseases has emerged in film recently. This trend corresponds to the current situation in Germany. The proportion of non-cancer patients who receive palliative care at the end of life is slowly but steadily increasing, as the awareness of end-of-life issues in this population is growing (Gemeinsamer Bundesausschuss 2012).

However, the portrayal of terminal illness in film often does not reflect the known symptomatology of terminal illness. Most of the symptoms from which the main characters suffer, such as hair loss and fever, can be attributed instead to the side-effects of anticancer treatments. The more characteristic symptoms of advanced cancer, such as cachexia, perhaps because their portrayal would be more difficult, are less often seen in film.

Noticeably, palliative and hospice care, important approaches for people suffering from terminal illnesses, were not mentioned by word at all in any of the analyzed movies. Though, some scenes were shown in which palliative or hospice care can be assumed, but is never named explicitly. This fact is even more surprising because palliative and hospice care have experienced a very dynamic development in past two decades nationally (Deutsche Gesellschaft für Palliativmedizin 2013) and internationally. This end-of-life care may have been overlooked because the movies did not focus on the imminently dying. Further, producers and directors might fear that the negative connotations and prejudices associated with palliative care might lead to lower attendance of their movies (Webster and Kristjanson 2002; Fadul et al. 2009). Lastly, structures of palliative and hospice care may still not be known well enough to be brought into the movies.

### Study limitations

Only a small number of international movie productions were considered in this descriptive analysis. Only a small minority of movies dealt with terminal illness and even fewer of these movies were ranked highly enough in ticket sales to garner recognition by the FFA and admittance to this study. Further, the movies ranked in the FFA hit lists derive their ticket sale numbers exclusively from German cinemas. However, this is a first systematic insight in to the issue of dying and death in popular movies, not an exhaustive one.

Due to the limited number of movies reviewed and the missing international viewer numbers, generalizability of this study to other movies or countries is limited and on that account this examination should be interpreted carefully as a descriptive study.

Additionally, it cannot be guaranteed, because of shortened or incomplete movie summaries, that all movies dealing with terminal illnesses and eligible for inclusion were detected during this examination.

## Conclusions

The number of movies dealing with terminal illness is increasing and a considerable audience has become interested in these films. The limited true-to-life performances observed may be in part explained by dramaturgical reasons, but a presentation closer to reality could be a major public educational resource. It could also help people concerned (patients/care-givers/health care professionals) with end-of-life issues brought about by terminal illness. Further, the presence of more accurate portrayals of palliative and hospice care on the big screen would serve to increase attention toward the field. The presentation of palliative and hospice care could be an effective way to eliminate prejudices and barriers that prevent a proper understanding of palliative care (Fadul et al. 2009; Miyashita et al. 2008).
